# The Activation of Reticulophagy by ER Stress through the ATF4-MAP1LC3A-CCPG1 Pathway in Ovarian Granulosa Cells Is Linked to Apoptosis and Necroptosis

**DOI:** 10.3390/ijms24032749

**Published:** 2023-02-01

**Authors:** Huiduo Li, Yanan Jing, Xiaoya Qu, Jinyi Yang, Pengge Pan, Xinrui Liu, Hui Gao, Xiuying Pei, Cheng Zhang, Yanzhou Yang

**Affiliations:** 1 Key Laboratory of Fertility Preservation and Maintenance, Ministry of Education, Key Laboratory of Reproduction and Genetics in Ningxia, Department of Histology and Embryology of Basic Medical College, Ningxia Medical University, Yinchuan 750004, China; 2 College of Life Science, Capital Normal University, Beijing 100048, China

**Keywords:** reticulophagy, ER stress, unfolded protein response, CCPG1, granulosa cells, necroptosis, apoptosis

## Abstract

Female infertility is caused by premature ovarian failure (POF), which is triggered by the endoplasmic reticulum (ER) stress-mediated apoptosis of granulosa cells. The ER unfolded protein response (UPR^er^) is initiated to promote cell survival by alleviating excessive ER stress, but cellular apoptosis is induced by persistent or strong ER stress. Recent studies have reported that reticulophagy is initiated by ER stress. Whether reticulophagy is activated in the ER stress-mediated apoptosis of granulosa cells and which pathway is initiated to activate reticulophagy during the apoptosis of granulosa cells are unknown. Therefore, the role of reticulophagy in granulosa cell death and the relationship between ER stress and reticulophagy were investigated in this work. Our results suggest that the ER stress inducer tunicamycin causes POF in mice, which is attributed to the apoptosis of granulosa cells and is accompanied by the activation of UPR^er^ and reticulophagy. Furthermore, granulosa cells were treated with tunicamycin, and granulosa cell apoptosis was triggered and increased the expression of UPR^er^ and reticulophagy molecules. The expression of ATF4 was then downregulated by RNAi, which decreased the levels of autophagy and the reticulophagy receptor CCGP1. Furthermore, ATF4 targets MAP1LC3A, as revealed by the ChIP sequencing results, and co-IP results demonstrated that MAP1LC3A interacts with CCPG1. Therefore, reticulophagy was activated by ER stress through the ATF4-MAP1LC3A-CCPG1 pathway to mitigate ER stress. Additionally, the role of reticulophagy in granulosa cells was investigated by the knockdown of CCPG1 with RNAi. Interestingly, only a small number of granulosa cells died by apoptosis, whereas the death of most granulosa cells occurred by necroptosis triggered by STAT1 and STAT3 to impair ER proteostasis and the ER protein quality control system UPR^er^. Taken together, the results indicate that the necroptosis of granulosa cells is triggered by up- and downregulating the reticulophagy receptor CCPG1 through STAT1/STAT3-(p)RIPK1-(p)RIPK3-(p)MLKL and that reticulophagy is activated by ER stress through the ATF4-MAP1LC3A-CCPG1 pathway.

## 1. Introduction

Premature ovarian failure (POF) is defined as the loss of ovarian function before the age of 40 years, and the follicles in the ovary rapidly decrease to no or few residual follicles in women younger than 40 years [1]; infertility is also caused by POF [2]. Ovarian atresia is caused by granulosa cell apoptosis and mainly contributes to POF [3,4].

The endoplasmic reticulum (ER) plays essential roles in protein folding, transport, and synthesis, but the microenvironment of the ER can be disturbed by ER stress [5,6] through Ca^2+^ deficiency, hypoxia, and N-terminal glycosylation dysfunction, and this disturbance is accompanied by an excessive accumulation of unfolded and misfolded proteins in the ER. Consequently, the ER unfolded protein response (UPR^er^) is initiated to alleviate ER stress and promote cell survival. Three pathways in the UPR^er^ have been identified: the PERK (pancreatic ER kinase)/eIF2α (eukaryotic initiation factor 2α)/ATF4 (activating transcription factor 4) pathway, the IRElα (inositol requiring enzyme 1α)/XBP-1 (X-box-binding protein) pathway, and the ATF6α (activating transcription factor 6α) pathway [7]. When the UPR^er^ is activated, the ER burden of unfolded and misfolded proteins is relieved by three processes [8]: ER-associated degradation (ERAD) and lysosome-mediated autophagy [9]. However, cellular apoptosis is initiated by excessive or persistent ER stress through the activation of the transcription factor CHOP and through the c-Jun N-terminal kinase (JNK) pathway and caspase-dependent pathways [10].

The role of ER stress has been largely explored in many studies and is involved in several physiological and pathological processes, including ovarian follicle development and atresia, early embryo development and death, embryo implantation, placenta formation, delivery, and fetal growth restriction (FGR) [10,11,12]. Additionally, our study revealed that ER stress is involved in several processes of female reproduction [10]; in particular, ovarian atresia and the apoptosis of granulosa cells are triggered by the ER stress-mediated apoptotic pathway [10].

Reticulophagy is a type of selective autophagy and is induced by ER stress [13]; unlike ER stress-induced autophagy, the activation of reticulophagy requires several receptors, including FAM134B, RTN3L, ATL3, SEC62, CCPG1, and TEX264, in mammals [14], and CCPG1 plays an important role in ER proteostasis. Reticulophagy mediates the selective degradation of the ER through the reticulophagy receptor [15]. Reticulophagy is a major quality control mechanism of the ER that maintains ER homeostasis and ER proteostasis [14]. Reticulophagy has emerged as a central component of the ER remodeling process [16]. Pathways of ER homeostasis and ER quality control alleviate ER expansion which is induced by ER stress and act as alternative disposal pathways for misfolded and fragmented proteins, which are delivered to the lysosome for degradation [17,18]. The dysfunction of reticulophagy might be connected with POF. The mechanism through which reticulophagy controls ER proteostasis and cellular homeostasis are unclear, and whether reticulophagy is induced by ER stress in ovarian atresia and granulosa cell apoptosis is largely unknown.

As a reticulophagy receptor, CCPG1 (cell cycle progression gene 1) is inducible by the UPR and links ER stress to reticulophagy through ATG8 family proteins [19]. In vivo, CCPG1 protects against ER luminal protein aggregation and consequent UPR hyperactivation and tissue injury of the exocrine pancreas [19]. CCPG1 plays an essential role in maintaining ER proteostasis, but CCPG1 loss sensitizes the exocrine pancreas to tissue injury by disturbing ER proteostasis [20]. ER stress causes an increase in CCPG1 transcripts, providing a direct link between ER stress and reticulophagy, and CCPG1 prevents the hyperaccumulation of insoluble proteins within the ER lumen of pancreatic acinar cells and alleviates ER stress [20]. Therefore, ER proteostasis and ER homeostasis are maintained by CCPG1 [21]. However, the role of CCPG1 in ovarian atresia and granulosa cell death is largely unknown.

ATG8/LC3 is essential for autophagosome biogenesis/maturation and also functions as an adaptor protein for selective autophagy pathways, such as reticulophagy. In mammalian cells, several homologues of yeast Atg8, including MAP1LC3 (A, B, C), GABARAP, and GABARAPL 1/2, have been identified [22]. ATF4 links ER stress with reticulophagy in glioblastoma cell death [23]. Whether ATF4 links ER stress and reticulophagy in ovarian atresia and granulosa cell death and how ATF4 is linked to CCPG1 is unclear.

Here, we explored the role of the reticulophagy receptor CCPG1 in ovarian follicle atresia and granulosa cell death and the interaction of ER stress and reticulophagy in ovarian atresia and granulosa cell death. This work provides a novel insight into reticulophagy in ovarian atresia and granulosa cell death and provides a novel drug target for ER dysfunction-induced granulosa cell death and ovarian atresia via reticulophagy.

## 2. Results

### 2.1. The Activation of Reticulophagy in the ER by the Stress Inducer Tunicamycin Causes POF

**Figure 1:**
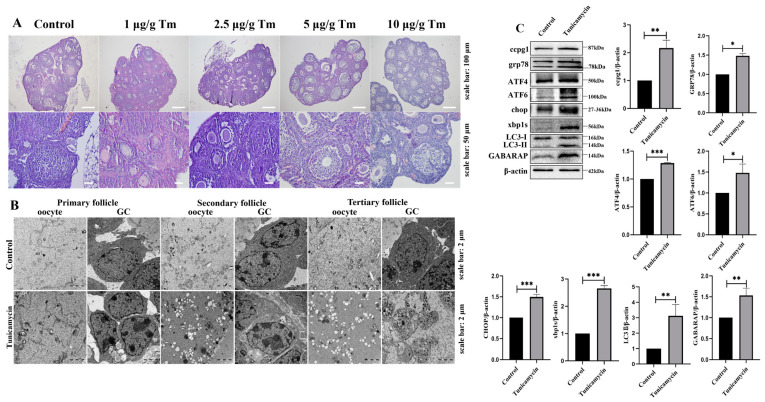
Reticulophagy activated by the ER stress inducer tunicamycin causes POF. (**A**): Histology of ER stress inducer tunicamycin (Tm)-treated mouse ovaries; (**B**): Ultrastructure of oocytes and granulosa cells (GCs) in primary, secondary, and tertiary follicles by transmission electron microscopy detection; (**C**): Western blot analysis of ovaries from control and 5 μg/g tunicamycin-treated mice. β-actin was used as a reference protein, *** *p* < 0.001, ** *p* < 0.01, * *p* < 0.05.

Our previous study revealed that the apoptosis of mouse granulosa cells was triggered by the ER stress inducer tunicamycin [4], and granulosa cell apoptosis is the main contributor to ovarian follicle atresia, which eventually leads to POF. Here, we found that POF was triggered by the ER stress inducer tunicamycin, and a more substantial initiation of POF was observed with 5 and 10 μg/g tunicamycin than with the control or 1 or 2.5 μg/g tunicamycin (Figure 1A). Subsequently, the ultrastructure of granulosa cells and oocytes from primary to tertiary follicles was observed by transmission electron microscopy (TEM). In 5 μg/g tunicamycin-treated mice, the ultrastructural alteration of granulosa cells and oocytes in primary follicles was not apparent, but the vacuolization and swelling of the mitochondria and ER were observed (Figure 1B), which might contribute to the death of granulosa cells and oocytes. Indeed, ER stress and the UPR^er^ pathway were activated, and marker molecules, including ATF4, ATF6, CHOP, and XBP1s, were significantly increased in mice with POF induced by 5 μg/g tunicamycin compared with the control mice. Interestingly, the reticulophagy pathway was also activated, and the marker molecules CCPG1, LC3II, and GABARAP were significantly increased in mice with POF induced by 5 μg/g tunicamycin compared with the control mice (Figure 1C). Therefore, reticulophagy was activated in ER stress-induced POF.

### 2.2. ER Stress Triggers Reticulophagy and Apoptosis of Granulosa Cells

**Figure 2:**
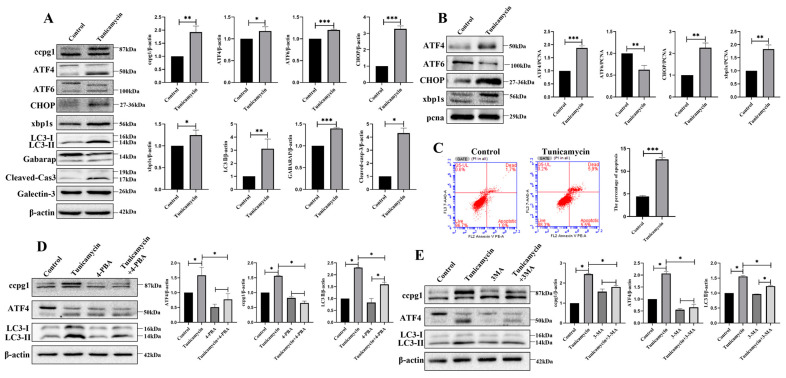
Reticulophagy activated by the ER stress inducer tunicamycin causes granulosa cell apoptosis. (**A**): Detection of the UPR^er^ and reticulophagy by Western blotting and analysis of total protein in the control and 5 μM tunicamycin-treated granulosa cells; (**B**): Detection of the UPR^er^ by Western blotting and analysis of nuclear protein in the control and 5 μM tunicamycin-treated granulosa cells; (**C**): Detection of cellular apoptosis by flow cytometry in the control and 5 μM tunicamycin-treated granulosa cells; (**D**): Detection of the UPR^er^ molecule ATF4 and reticulophagy by Western blotting and analysis of total protein in the control, 5 μM tunicamycin-, 5 Mm+4-PBA-, and 5 μM tunicamycin+5 mM 4-PBA-treated granulosa cells; (**E**): Detection of the UPR^er^ molecule ATF4 and reticulophagy by Western blotting and analysis of total protein in the control, 5 μM tunicamycin-, 10 mM 3-MA-, and 5 μM tunicamycin+10 mM 3-MA-treated cells. β-actin was used as a reference protein, *** *p* < 0.001, ** *p* < 0.01, * *p* < 0.05.

The apoptosis of granulosa cells is the main contributor to follicle atresia and leads to POF. ER stress was initiated by the inducer tunicamycin. In 5 μM tunicamycin-treated granulosa cells, the UPR^er^ marker molecules ATF4, ATF6, CHOP, and XBP1s and the reticulophagy marker molecules CCPG1, LC3II, and GABARAP were increased, and these increases were accompanied by an increase in the apoptotic marker cleaved caspase-3 (Figure 2A); therefore, reticulophagy and cell apoptosis were activated by ER stress. Furthermore, in tunicamycin-treated cells, the UPR^er^ was activated, and this activation was accompanied by the increased nuclear protein levels of ATF4, CHOP, and XBP1s but decreased nuclear protein levels of ATF6 (Figure 2B).

Consistent with the Western blot results, the apoptosis rate of granulosa cells was significantly higher in tunicamycin-treated granulosa cells than in the control cells (Figure 2C). Therefore, ER stress triggers the reticulophagy and apoptosis of granulosa cells. Furthermore, ER stress was inhibited by the ER stress inhibitor 4-PBA (4-phenylbutyric acid) and the autophagy inhibitor 3-MA (3-methyladenine), and the results showed that ATF4, CCPG1, and LC3II were all decreased in 4-PBA+tunicamycin- and 3-MA+tunicamycin-treated granulosa cells compared with tunicamycin-treated granulosa cells (Figure 2D,E). These results emphasize that the UPR^er^ marker molecule ATF4 is positively related to reticulophagy and that ATF4 links ER stress with reticulophagy.

**Figure 3:**
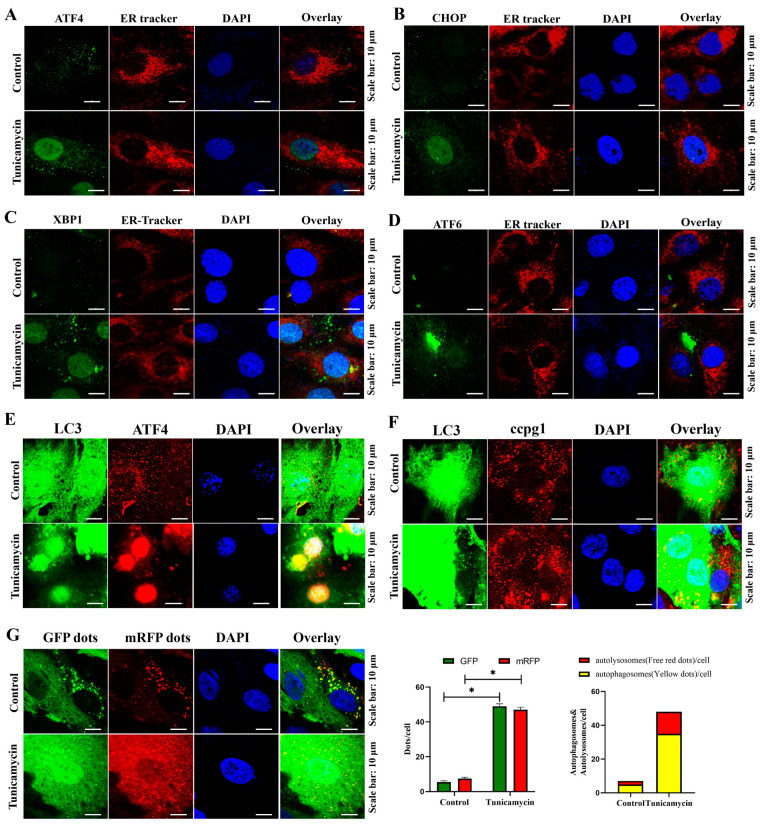
Immunofluorescence of markers of the UPR^er^ and autophagy in control and 5 μM tunicamycin-treated cells. (**A**): Colocalization of ATF4 (green) protein and ER tracker (red); (**B**): Colocalization of CHOP (green) protein and ER tracker (red); (**C**): Colocalization of XBP1s (green) protein and ER tracker (red); (**D**): Colocalization of ATF6 (green) protein and ER tracker (red); (**E**): Colocalization of ATF4 (red) protein and GFP-LC3 (green); (**F**): Colocalization of CCPG1 (red) protein and GFP-LC3 (green); (**G**): Autophagic flux detection by mRFP-GFP-LC3B staining. At least 50 different cells from different fields were analyzed, and the dots were blindly counted by three different persons. The nuclei were stained with DAPI (blue). * *p* < 0.05.

Additionally, the localization of transcription factors in the UPR^er^ pathway was assessed. Consistent with the Western blot results, the transcription factors ATF4 (Figure 3A), CHOP (Figure 3B), and XBP1s (Figure 3C), but not ATF6 (Figure 3D), were translocated into the nucleus during the ER stress-mediated apoptosis of granulosa cells; hence, the UPR^er^ was activated to maintain ER proteostasis. In addition, further evidence showing an increase in the colocalization of ATF4 and GFP-LC3B in the nucleus (Figure 3E) and GFP-LC3B and CCPG1 in the cytoplasm (Figure 3E) and an increased autophagic flux (Figure 3G) in tunicamycin-treated granulosa cells indicated that reticulophagy was activated in ER stress-mediated apoptosis. Therefore, ATF4 bridges ER stress and reticulophagy.

### 2.3. ATF4 Targets MAP1LC3A to Activate the Reticulophagy Receptor CCPG1

**Figure 4:**
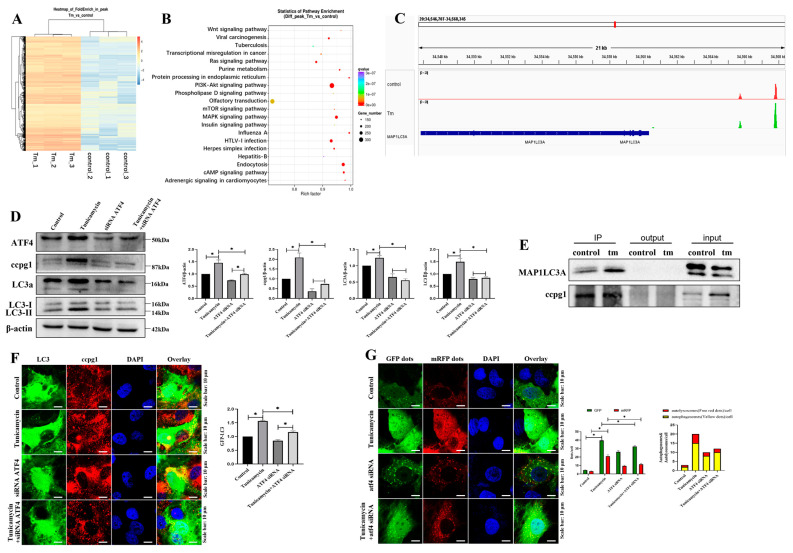
ATF4 targets MAP1LC3/CCPG1. (**A**): Heatmap of control and 5 μM tunicamycin-treated cells generated from the ChIP sequencing results; (**B**): Enriched KEGG pathway analysis of the ChIP sequencing results; (**C**): Binding site of ATF4 in MAP1LC3A; (**D**): Detection of reticulophagy by Western blotting and results of ATF4 inhibition in 5 μM tunicamycin-treated cells; (**E**): MAP1LC3A interacts with CCPG1, as revealed by co-IP; (**F**): Colocalization of CCPG1 (red) protein and GFP-LC3 (green); (**G**): Autophagic flux detection by mRFP-GFP-LC3B staining. The nuclei stained with DAPI (blue). At least 50 different cells in different fields were analyzed, and the dots were blindly counted by three different individuals. * *p* < 0.05.

The potential target of ATF4 in ER stress-induced granulosa cell apoptosis was confirmed by ChIP-sequencing, and the heatmap results revealed that ATF4 target genes were markedly altered in tunicamycin-treated granulosa cells compared with the control cells (Figure 4A). Furthermore, the target genes were evaluated by KEGG pathway enrichment analysis, and the top 20 enriched pathways included the mTOR signaling pathway, PI3K-AKT signaling pathway, and MAPK signaling pathway (Figure 4B). In addition to the top 20 enriched pathways, the regulation of the autophagy pathway was identified, and ATF4 targeted 22 autophagic genes, including ATG16L1, ATG10, ATG7, ATG5, and MAP1LC3A (Supplementary Table S1). MAP1LC3A belongs to the ATG8 family and is involved in reticulophagy [19]. ATF4 binds to the reticulophagy protein CCPG1 via MAP1LC3A, and this result was further supported by the binding site analysis, which showed that ATF4 is bound to the promoter of MAP1LC3A (Figure 4C). Furthermore, CCPG1, LC3II, and MAP1LC3A were all decreased in ATF4-knockdown cells compared with the control cells, and CCPG1, LC3II, and MAP1LC3A were all decreased in ATF4 siRNA- and tunicamycin-treated cells compared with tunicamycin-treated cells (Figure 4D). These results further emphasize that CCPG1, LC3II, and MAP1LC3A are regulated by ATF4.

Reticulophagy was activated by autophagy combined with the reticulophagy receptor, and according to our coimmunoprecipitation (co-IP) results, MAP1LC3A directly interacted with CCPG1 (Figure 4E). These findings were further supported by our observation of the markedly decreased colocalization of CCPG1 and GFP-LC3B (Figure 4F) and decreased autophagic flux (Figure 4G) in ATF4 siRNA and tunicamycin-treated cells compared with tunicamycin-treated cells.

Hence, ER stress activates reticulophagy via the ATF4/MAP1LC3A/CCPG1 pathway, and this effect is accompanied by cell apoptosis.

### 2.4. Impaired ER Proteostasis in CCPG1-Deficient and CCPG1-Overexpressing Cells

**Figure 5:**
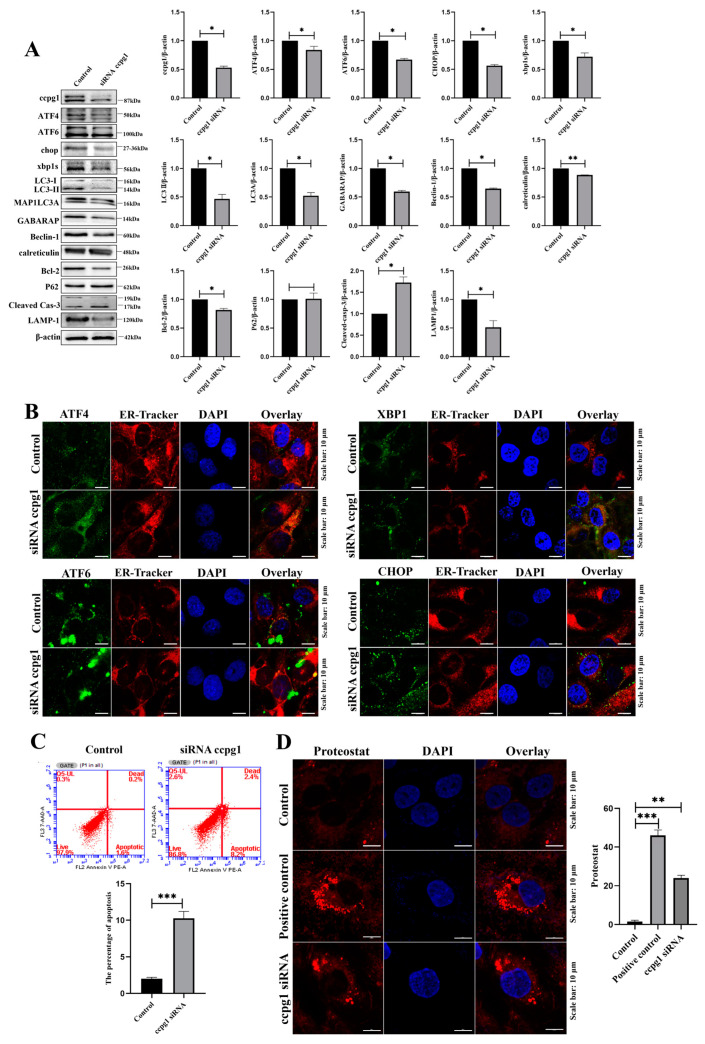
Impaired ER proteostasis in CCPG1-knockdown cells. (**A**): Detection of the UPR^er^, reticulophagy and apoptotic marker molecules by Western blotting in CCPG1-knockdown granulosa cells; (**B**): Colocalization of ATF4 (green), CHOP (green), XBP1s (green), ATF6 (green), and ER tracker (red) in CCPG1-knockdown granulosa cells; (**C**): Detection of cellular apoptosis by flow cytometry in the control and CCPG1-knockdown cells; (**D**): Protein aggregate detection results. At least 50 different cells in different fields were analyzed, and the dots were blindly counted by three different individuals. β-Actin was used as a reference protein, *** *p* < 0.001, ** *p* < 0.01, * *p* < 0.05. The nuclei are stained with DAPI (blue).

The protein quality control (PQC) machinery of the UPR plays a critical role in the selective identification and removal of mistargeted, misfolded, and aberrant proteins [24]. The UPR transmits information about the protein folding status to the nucleus and cytosol to adjust the protein folding capacity of the cell [25], and ER proteostasis is mediated by the UPR^er^ [25]. Therefore, the UPR^er^ pathway was examined, and the results suggested that the expression levels of the UPR^er^ marker molecules ATF4, ATF6, CHOP, and XBP1s were all decreased in CCPG1-knockdown cells compared with the control cells (Figure 5A). This result suggests that the impairment of the UPR^er^ occurs, leading to the dysfunction of the ER, which is attributed to aggregated unfolded and misfolded proteins in the ER, and that the dysfunctional ER is then cleared by the reticulophagy-lysosome system.

The lysosomal degradation pathway of autophagy plays a fundamental role in cellular, tissue, and organismal homeostasis [26], and ER homeostasis is maintained by reticulophagy [27]. Our results revealed that reticulophagy was inhibited due to a decreased expression of the reticulophagy marker molecules LC3II, MAP1LC3A, GABARAP, and Beclin1 and decreased expression of the lysosome marker molecule lamp1 in CCPG1-knockdown cells compared with the control cells (Figure 5A). These results suggest that the reticulophagy-lysosome system was also disturbed. Additionally, the colocalization of four transcription factors in the UPR^er^ pathway and ER-tracker was detected. Interestingly, the four transcription factors ATF4, CHOP, XBP1s, and ATF6 all aggregated in the ER but did not translocate into the nucleus (Figure 5B). These results further indicate that the dysfunction of the UPR^er^ and reticulophagy was accompanied by an increased apoptosis rate in granulosa cells (Figure 5C). Furthermore, protein aggregates were markedly increased in CCPG1-knockdown cells compared with the control cells (Figure 5D). Additionally, consistent with the results observed in CCPG1-knockdown cells, the nuclear protein levels of ATF4, ATF6, CHOP, and XBP1s were all significantly decreased in CCPG1-overexpressing cells compared with the control cells (Supplementary Figure S1A), and this result was supported by the localization of ATF4, CHOP, XBP1s, and ATF6, and four transcription factors did not translocate into the nucleus (Supplementary Figure S1B). Therefore, the UPR^er^ was impaired, ER proteostasis was disturbed in CCPG1-knockdown and CCPG1-overexpressing cells, and ER proteostasis was maintained by moderate levels of CCPG1.

### 2.5. The Up- and Downregulation of CCPG1 Triggers Necroptosis of Granulosa Cells

To investigate the role of the reticulophagy receptor CCPG1, the proteome was assessed using a iTRAQ method, and the results presented in a heatmap suggested that the protein expression was markedly altered in CCPG1 siRNA-treated cells compared with the control cells (Supplementary Figure S2A). The results of the volcano map revealed that 612 proteins exhibited an increased expression, and 185 proteins exhibited a decreased expression (Supplementary Figure S2B). Furthermore, the differentially expressed proteins (DEPs) were evaluated by KEGG pathway enrichment analysis; the results showed that 797 DEPs were enriched in 316 pathways (Supplementary Table S2), and some pathways related to cell death, such as apoptosis, autophagy, and necroptosis, were identified (Supplementary Figure S2C).

**Figure 6:**
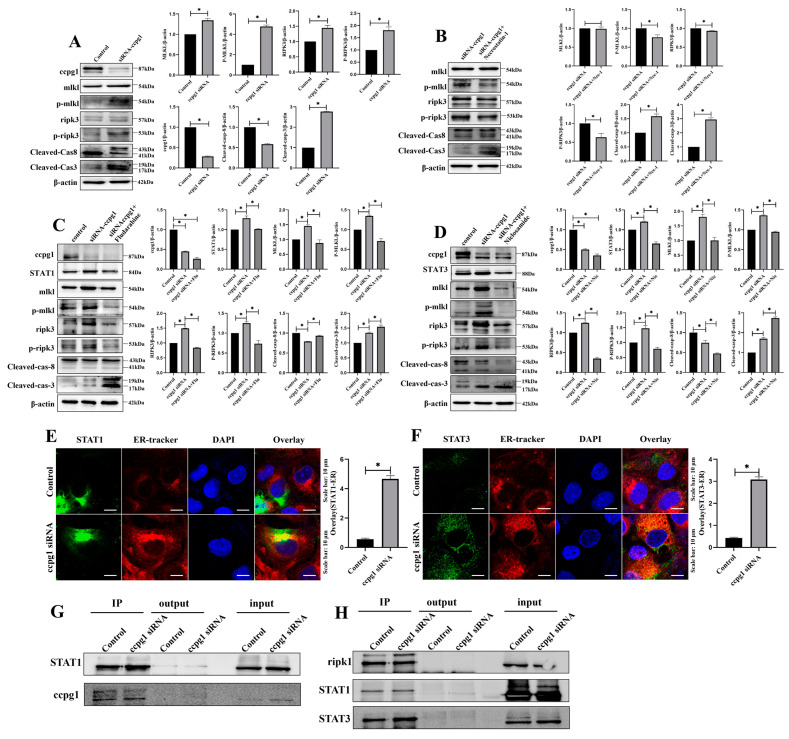
Activation of necroptosis by downregulation of CCPG1 by STAT1 and STAT3. (**A**): Results from the detection of necroptosis by Western blotting in CCPG1-knockdown granulosa cells; (**B**): Detection of necroptosis by Western blotting in granulosa cells treated with the necroptosis inhibitor necrostatin-1; (**C**): Detection of necroptosis and STAT1 by Western blotting in granulosa cells treated with the STAT1 inhibitor fludarabine; (**D**): Detection of necroptosis and STAT1 by Western blotting in granulosa cells treated with the STAT3 inhibitor niclosamide; (**E**): Colocalization of STAT1 (Green) and ER tracker (Red) in CCPG1-knockdown cells; (**F**): Colocalization of STAT3 (Green) and ER tracker (Red) in CCPG1-knockdown cells; (**G**): CCPG1 directly interacts with STAT1, as revealed by co-IP detection; (**H**): STAT1 and STAT3 directly interact with ripk1, as revealed by co-IP detection. β-Actin was used as a reference protein, * *p* < 0.05.

Autophagy is a double-edged sword in cells, and cell homeostasis is maintained with baseline autophagy [28]. Therefore, the effect of reticulophagy on granulosa cells was investigated through the up- and downregulation of CCPG1 expression. Interestingly, granulosa cell necroptosis was triggered when the expression of CCPG1 was up- and downregulated, and the increased expression of the necroptosis marker molecules p-ripk3 and p-mlkl and decreased expression of cleaved caspase-8 were found in these cells compared with the control cells (Figure 6A and Figure S3A). Additionally, necroptosis was reversed by the necroptosis inhibitor necrostatin-1 in cells in which CCPG1 was up- and downregulated, and this reversal was accompanied by the decreased expression of the necroptosis marker molecules p-ripk3 and p-mlkl and increased expression of cleaved caspase-8 (Figure 6B and Figure S3B). In addition, the apoptosis of granulosa cells was significantly increased in cells in which CCPG1 was up- and downregulated compared with the control cells (Figure 6A and Figure S3A), as determined by the expression of cleaved caspase-3, but apoptosis was not inhibited by the necroptosis inhibitor necrostatin-1 (Figure 6B and Figure S3B). Therefore, the apoptosis of granulosa cells initiated by the up- and downregulation of CCPG1 expression was independent of necroptosis, and cell survival was maintained by a baseline level of reticulophagy. Cell necroptosis was also triggered by deficient and excessive reticulophagy.

### 2.6. CCPG1 Triggers Necroptosis of Granulosa Cells via STAT1 and STAT3

To explore the mechanism of CCPG1-induced necroptosis, two proteins in the necroptosis pathway, STAT1, and STAT3, were observed in the proteome, and we speculated that CCPG1 triggered necroptosis via STAT1 and STAT3. Consistently, STAT1 and STAT3 were both increased in GGPG1 siRNA-treated granulosa cells compared with control cells, and this increase was accompanied by increased necroptosis (Figure 6C,D); in addition, the expression levels of STAT1 and STAT3 were reduced by the inhibitors fludarabine and niclosamide, respectively, and the expression levels of the necroptosis marker molecules p-ripk3 and p-mlkl were decreased, and the expression level of cleaved caspase-8 was increased in fludarabine-treated and niclosamide-treated CCPG1-knockdown cells compared with untreated CCPG1-knockdown cells (Figure 6C,D). Necroptosis was significantly decreased in CCPG1-knockdown cells when cotreated with fludarabine and niclosamide compared with fludarabine-treated CCPG1-knockdown cells and niclosamide-treated CCPG1-knockdown cells, and this decrease was accompanied by decreased p-ripk3 and p-mlkl levels and increased cleaved caspase-8 levels (Supplementary Figure S4). Furthermore, the colocalization of the STAT1, STAT3, and ER tracker was markedly increased in CCPG1-knockdown cells compared with the control cells (Figure 6E,F), and CCPG1 directly interacts with STAT1 (Figure 6G). STAT1 and SATA3 directly target ripk1 (Figure 6H), which is the key upstream molecule for necroptosis [29,30]. Therefore, the knockdown of CCPG1 induces cell necroptosis through the STAT1/STAT3-(p)RIPK1-(p)RIPK3-(p)MLKL pathway.

### 2.7. ER Stress Induced Apoptosis Negatively with Necroptosis

To explore the relationship between ER stress-induced apoptosis and necroptosis, necroptosis was found to be reduced in cells undergoing ER stress-induced cellular apoptosis, and this reduction was accompanied by the decreased expression of p-ripk3 and p-mlkl, increased expression of cleaved caspase-8, and of the apoptotic marker molecule cleaved caspase-3 compared with the levels observed in control cells (Supplementary Figure S5A). Furthermore, the reduction in ER stress-mediated apoptosis by the ER stress inhibitor 4-PBA increased necroptosis, and this increase was accompanied by the increased expression of p-ripk3 and p-mlkl in cells cotreated with 4-PBA and tunicamycin compared with tunicamycin-treated cells (Supplementary Figure S5B). Therefore, ER stress-induced apoptosis was negatively correlated with necroptosis.

## 3. Discussion

The unfolded stress response (UPR) is a conserved cellular pathway involved in PQC to maintain homeostasis under cell stress [31]. When ER stress occurs, the UPR^er^ is triggered to mitigate ER stress and restore ER homeostasis for the promotion of cell survival, but cell apoptosis induced by excessive or persistent ER stress and the ER burden is alleviated through the UPR^er^ pathway [10]. If the ER burden of the misfolded and unfolded proteins is not cleared by the UPR^er^, autophagy or selective autophagy-reticulophagy is initiated to maintain ER proteostasis and ER homeostasis. Therefore, continuous ER stress and the UPR can activate reticulophagy to coordinately regulate the dynamic structure and critical functions of the ER [32]. Indeed, several studies have found that reticulophagy is activated by ER stress through the UPR^er^ [13,33,34], but the results of those studies did not reveal whether reticulophagy was activated by ER stress in ovarian follicle development and atresia via the UPR^er^.

Our results revealed that ovarian follicle atresia was triggered by ER stress-mediated cellular apoptosis, eventually leading to POF, and these effects were accompanied by UPR^er^ activation and reticulophagy. Consistently, ER stress and the UPR^er^ are involved in ovarian follicle atresia by triggering the apoptosis of granulosa cells [35,36,37], and ER stress and the UPR^er^ contribute to ovarian aging [38] and POF [39].

ER stress perturbs the ER function, further initiating the UPR and ER stress-associated autophagy to re-establish the ER equilibrium; otherwise, unresolved ER stress is bound to cause cell death by inducing apoptosis [40,41]. If the ER burden is unloaded through the UPR^er^ pathway, the protein aggregate and dysfunctional ER are cleared by ER-associated degradation (ERAD), which has two forms: ubiquitin/proteasome ERAD(I) and autophagy/lysosome ERAD(II) [42]. Our results suggested that autophagy and selective autophagy-reticulophagy were both initiated during ER stress and UPR^er^-induced ovarian follicle atresia in vivo and granulosa cell apoptosis in vitro. Therefore, reticulophagy was activated by ER stress in granulosa cell apoptosis and ovarian follicle atresia. Indeed, reticulophagy is initiated by ER stress in several cell types [43,44,45]; thus, the UPR^er^ might be a bridge between reticulophagy and ER stress [13]. Furthermore, ER stress is linked with reticulophagy in glioblastoma cells via ATF4 [23], which is a key component of the UPR^er^ pathway and ovarian follicle development. Therefore, we speculate that ATF4 links ER stress and reticulophagy in granulosa cell apoptosis and ovarian follicle atresia. Furthermore, evidence from ChIP-sequencing has revealed that ATF4 directly binds to the promoter of MAP1LC3A, which is an ATG8 member of the mammalian family [22], and the decreased expression of CCPG1, LC3II, and MAP1LC3A in ATF4-downregulated cells further indicates that ATF4 targets MAP1LC3A during ER stress-induced reticulophagy.

Reticulophagy is initiated by the direct binding of autophagy receptors to ATG8 proteins [19]. Autophagy receptors recognize the cargo and/or define the organelle or organelle compartment to be degraded and interact with the autophagy modifier proteins of the Atg8/LC3 (light chain 3)/GABARAP (γ-aminobutyric acid receptor-associated protein) family via an Atg8-interacting motif (AIM) in yeast and via an LC3-interacting region (LIR) in mammals [46,47]. Our co-IP results indicate that MAP1LC3A directly binds to the reticulophagy receptor CCPG1, which is a receptor for ER stress-induced reticulophagy [19]. CCPG1 is a noncanonical autophagy cargo receptor that is essential for reticulophagy and ER proteostasis [48], and CCPG1 is a transmembrane ER receptor that functions in response to ER stress signals [18]. Therefore, ER stress activates reticulophagy through the ATF4/MAP1LC3A/CCPG1 pathway, and this effect is accompanied by increased cellular apoptosis. In the absence of ER stress, CCPG1 localizes to the perinuclear ER and in small foci at the ER periphery and contains an LIR at the N-terminal cytosolic domain engaging LC3/GABARAP [49]. During ER stress, endogenous CCPG1 is induced and drives the autophagic degradation of the peripheral ER [49]. Therefore, increased reticulophagy through the ATF4/MAP1LC3A/CCPG1 pathway alleviates ER stress and ER stress-mediated apoptosis.

Selective autophagy is one of the major forms of autophagy, which targets the degradation of specific substrates, particularly dysfunctional or superfluous organelles [50]. Reticulophagy, another type of autophagy, might be a double-edged sword, and our results show that ER proteostasis is maintained by moderate levels of reticulophagy. During ER stress, cells increase the synthesis of CCPG1 and promote reticulophagy to attenuate stress [19], and the expression level of CCPG1 is upregulated to drive the flux of peripheral reticulophagy [51]. CCPG1 is involved in the maintenance of ER protein homeostasis [19]. Insufficient CCPG1 causes the accumulation of protein aggregates and the loss of proteostasis [51]. Hence, CCPG1 plays an important role in ER proteostasis. In accordance with our hypothesis, cell proteostasis, including ER proteostasis, was disturbed by the downregulation of CCPG1, and this effect was attributed to the dysfunction of the UPR^er^ accompanied by increased protein aggregation. Furthermore, the UPR^er^ and reticulophagy were decreased, and the inhibition of the UPR^er^ and reticulophagy led to disturbed ER proteostasis and ER dysfunction. In addition, the levels of cell necroptosis-related molecules, such as STAT1 and STAT3, were increased. The expression levels of the necroptosis marker molecules (p-ripk3 and p-mlkl) were both increased in CCPG1-knockdown cells; interestingly, increased cell necroptosis was also observed in CCPG1-overexpressing cells. Therefore, the development of granulosa cells and ovarian follicles was controlled by moderate levels of reticulophagy, and the inhibition or hyperactivation of reticulophagy caused granulosa cell necroptosis and ovarian follicle atresia. Indeed, granulosa cell necroptosis is triggered by stress and subsequently starves oocytes, resulting in an increased vulnerability to ovarian follicle atresia and eventually leading to POF [52]. Therefore, necroptosis triggers ovarian follicle development [53,54]. Constitutive ER clearance maintains the volume of the organelle under normal growth conditions [55]. To uncover the pathway through which CCPG1 initiates necroptosis, this study revealed that necroptosis was reduced by the downregulation of the expression of STAT1 and STAT3 with specific inhibitors, and these results provide strong evidence showing that CCPG1 regulates necroptosis via STAT1 and STAT3. In addition to necroptosis, cell apoptosis was initiated in a small portion of CCPG1-knockdown cells but was not inhibited by a necroptosis inhibitor and was not dependent on necroptosis. Interestingly, apoptosis was increased by STAT1 and STAT3 inhibitors; therefore, both necroptosis and apoptosis were mediated by STAT1 and STAT3. Nevertheless, necroptosis was reduced in cells undergoing ER stress-mediated apoptosis accompanied by increased reticulophagy, and the inhibition of ER stress-induced apoptosis increased necroptosis and decreased reticulophagy; therefore, ER stress-mediated apoptosis is negatively related to necroptosis. We speculate that the increased expression of CCPG1 and decreased necroptosis improved reticulophagy to mitigate ER stress. Indeed, cells increase the synthesis of CCPG1 to promote reticulophagy and attenuate ER stress [19]. In addition, the UPR^er^ was activated in ER stress-induced POF and granulosa cell apoptosis, but CCPG1-induced necroptosis involves dysfunctions in ER proteostasis and UPR^er^.

In addition, the role of ER stress in oocyte was explored, and ER stress and UPR^er^ were involved in the oocyte maturation; hence, the attenuation of ER stress and UPR^er^ signaling plays an important role in oocyte maturation in vitro [56,57]. The induction of ER stress in the oocyte before in vitro fertilization causes lower fertilization rates and poor embryo development [58]. However, whether reticulophagy is also activated during ER stress in oocyte development is worthy of investigation.

**Figure 7:**
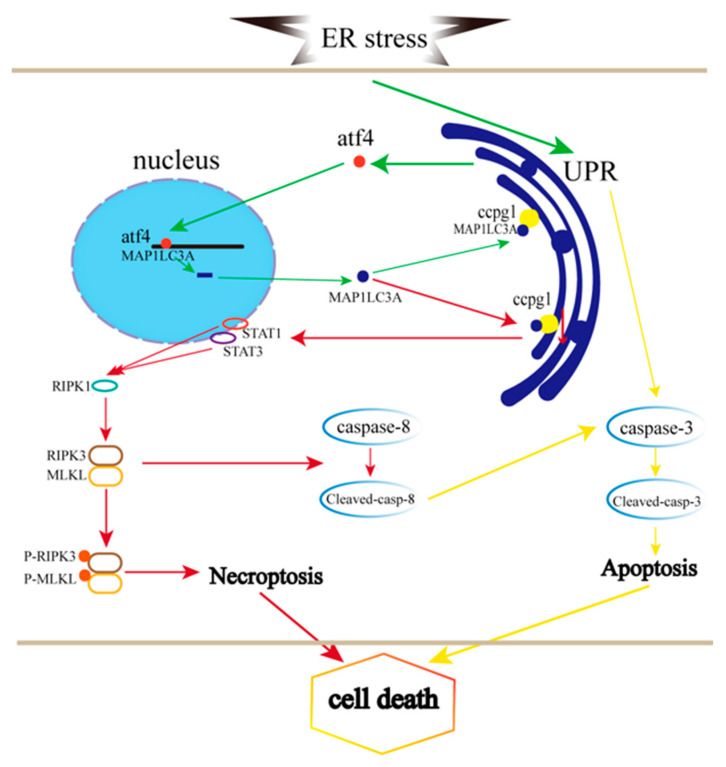
Pathway through which CCPG1 in necroptosis is linked to ER stress-mediated apoptosis. The green line indicates ER stress, the UPR^er^, and autophagy; the red line indicates necroptosis; the yellow line indicates apoptosis.

Taken together, the results demonstrate that granulosa cell necroptosis is caused by the reticulophagy receptor CCPG1 through the STAT1/STAT3-(p)RIPK1-(p)RIPK3-(p)MLKL pathway, which contributes to the impairing of ER proteostasis and the UPR^er^, and the ATF4/MAP1LC3A/CCPG1 pathway is activated to alleviate ER stress (Figure 7).

## 4. Materials and Methods

### 4.1. Animals

Twenty-one to 26-day-old mice were purchased from the laboratory animal center, bred in an animal facility at Ningxia Medical University, and maintained at 24 ± 2 °C in a light-controlled room (12 h light:12 h darkness) with free access to food and water. The animal procedures strictly followed the guidelines of the National Institutes of Health and were approved by the Institutional Animal Care and Use Committee at Ningxia Medical University. All the animals were operated on under sodium pentobarbital anesthesia, and all efforts were made to minimize pain and discomfort.

The mice were administered 1, 2.5, 5, and 10 μg/g tunicamycin, and the ovaries were collected after 24 h for histology and protein detection.

### 4.2. Inhibitors and Concentration

The ER stress inducer tunicamycin (T7765) was purchased from Sigma (Louis, MO, USA), and the concentration used for cell treatment was 5 μM according to a previous study [59]. The ER stress inhibitor 4-phenylbutyric acid (4-PBA, HY-A0281), the autophagy inhibitor 3-methyladenine (3-MA, HY-19312), the necroptosis inhibitor necrostatin-1 (HY-15760), the STAT1 inhibitor fludarabine (HY-B0069), and the STAT3 inhibitor niclosamide (HY-B0497) were purchased from MedChemExpress (New Jersey, USA), and the concentrations that were used were 5 mM [60], 10 mM [61], 20 μM [62], 50 μM [63], and 20 μM [64], respectively, according to previous studies.

### 4.3. Knockdown of ATF4 and CCPG1 in Granulosa Cells

ATF4 and CCPG1 expression was knocked down by RNAi using the RNAi sequences described previously [65,66]. For transfection, the human ganulosa cell line KGN was cultured in a DMEM/F12 medium with 10% FBS according to our previous study [67], and cells were seeded overnight to reach a cell concentration of 50–70%. siRNAs (GenePharma, Shanghai, China) and Lipofectamine 3000 (L3000015, Thermo Fisher, Waltham, MA, USA) were co-transfected into cells, according to the manufacturer’s instructions. Seventy-two hours later, the cells were harvested for detection.

For the overexpression of CCPG1, the coding sequence (CDS) was cloned into a lentivirus vector (lentiviral particles produced by Cyagen Biosciences (Suzhou, China)), and the cells were transfected with lentiviral particles (MOI 30) and polybrene (5–10 µg/mL). After 72 h, the cells were collected for detection.

### 4.4. Western Blot

Western blotting was performed according to our previous study [68]. Primary antibodies against MAP1LC3A (ab52628, RRID: AB_881227), CHOP (ab11419, RRID: AB_298023), XBP1s (ab220783, RRID: AB_2920809), ATF4 (ab184909, RRID: AB_2819059), Gabarap (ab109364, RRID: AB_10861928), PCNA (ab29, RRID: AB_303394), calreticulin (ab92516, RRID: AB_10562796), p-ripk3 (ab187091, RRID: AB_2619685), mlkl (ab184718, RRID: AB_2755030), p-mlkl (ab195117, RRID: AB_2768156), STAT3 (ab32500, RRID: AB_2286741) and GRP78 (ab32618, RRID: AB_732737) were purchased from Abcam; primary antibodies against ATF6 (24169-1-AP, RRID: AB_2876891), β-actin (20536-1-AP, RRID: AB_10700003), CCPG1 (13861-1-AP, RRID: AB_2074010), and STAT1 (10144-2-AP, RRID: AB_2286875) were purchased from Proteintech; primary antibodies against cleaved-caspase-3 (9661, RRID: AB_2341188), and cleaved-caspase-8 (9496, RRID: AB_561381) were purchased from Cell Signalling; and a primary antibody against LC3B (L7543, RRID: AB_796155) was purchased from Sigma (Louis, MO, USA). The secondary antibodies anti-rabbit IgG and anti-mouse IgG were purchased from Cell Signalling. β-Actin was used as a reference protein, and the protein bands were analyzed using ImageJ software.

### 4.5. ChIP Sequencing

ChIP sequencing and bioinformatic analyses were performed using Novogene (Beijing, China). Briefly, 10^6^ KGN cells in the control and tunicamycin-treated groups were harvested and crosslinked with formaldehyde. The cells were then lysed and sonicated, and an anti-ATF4 antibody was used for immunoprecipitation (IP). This step was followed by water bath decrosslinking, DNA purification, the construction of a sequencing library, amplification, and high-throughput sequencing. To identify the mechanisms underlying the role of ATF4 in regulating the target molecule in KGN cells, chromatin immunoprecipitation was performed using an ATF4 antibody followed by sequencing (ChIP-seq). Gene ontology (GO) analysis of differentially expressed genes (DEGs) and a Kyoto encyclopedia of genes and genomes (KEGG) pathway enrichment analysis of the captured downstream target genes was performed.

### 4.6. Proteomic Profiling

iTRAQ proteomics was used to comparatively quantify the proteome of CCPG1-knockdown cells and the control cells using the data analyzed by MajorBioLab Co., Ltd. (Shanghai, China). A total of 612 proteins displayed a greater than or equal to 1.2-fold increase in expression, and 185 proteins displayed a less than or equal to 0.83-fold decrease in expression in comparison to the control cells. The 797 DEPs were annotated based on their functional classification and functional enrichment. Enrichment analyses were separately performed with each quantile, and the overrepresented annotations were clustered through one-way hierarchical clustering for comparative analysis.

### 4.7. Immunofluorescence

The immunofluorescence procedure was performed according to our previous study [4]. The primary antibodies were used as described for Western blotting, and goat anti-rabbit FITC and goat anti-mouse TRITC secondary antibodies were purchased from Thermo Fisher. ER-Tracker Red (C1041) was purchased from Beyotime Biotechnology (Shanghai, China); a cell autophagy fluorescent detection kit (G0170) was purchased from Solarbio Life Sciences (Beijing, China); and Ad-GFP-LC3 (HBAD-1006) and Ad-mRFP-GFP-LC3 (HB-AP210 0001) were purchased from HANBIO (Shanghai, China). The images were obtained by confocal laser scanning microscopy (CLSM), and the fluorescence intensity was analyzed using ImageJ software.

### 4.8. Coimmunoprecipitation

The co-IP procedure was performed according to our previous study [68]. The antibodies used for co-IP were the same as those described for Western blotting.

### 4.9. Statistical Analyses

All experiments were repeated at least 3 times in triplicate each time. The data are presented as the means ± SEMs. The data were analyzed by ANOVA followed by Fisher’s least significant difference test and by a t-test using SPSS software (version 21.0; SPSS, Inc., Chicago, IL, USA). Differences were considered significant if *p* < 0.05.

## Data Availability

The datasets used and/or analyzed during the current study are available from the corresponding author upon reasonable request.

## Supplementary Materials

The following supporting information can be downloaded at: https://www.mdpi.com/article/10.3390/ijms24032749/s1.
