# Supplementary material for: The Activation of Reticulophagy by ER Stress through the ATF4-MAP1LC3A-CCPG1 Pathway in Ovarian Granulosa Cells Is Linked to Apoptosis and Necroptosis
Source: Int J Mol Sci. 2023 Feb 1;24(3):2749. doi: 10.3390/ijms24032749 (PMC9917250; doi:10.3390/ijms24032749)
Supplement: Supplementary file 1 [file ijms-24-02749-s001.zip › ijms-2100627-Supplementary.pdf]

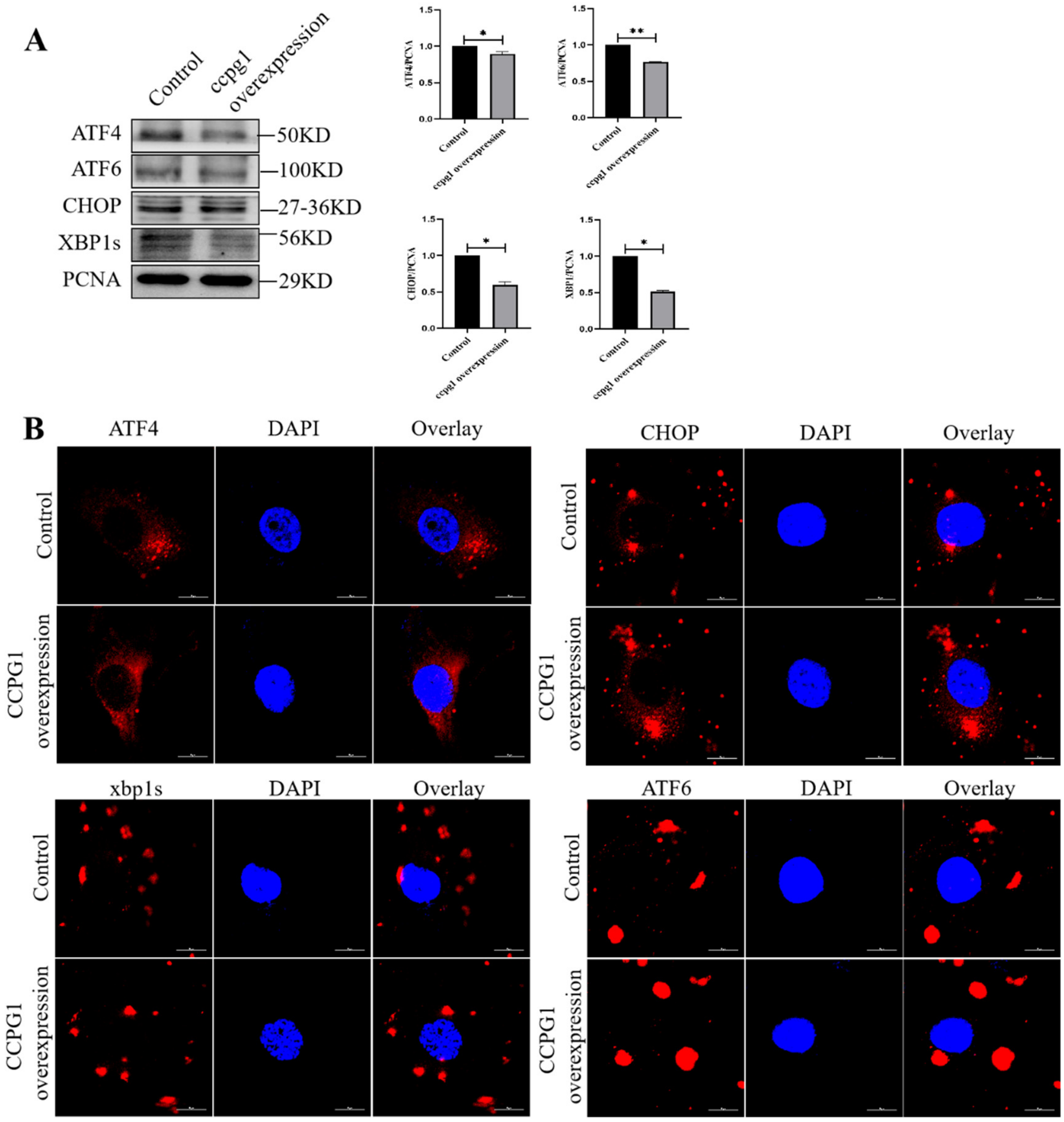

Figure S1. The impaired UPR^{er} in ccpg1 overexpression cells

A: Detection nuclear protein in UPR^{er} by western blot and results in over-expression of ccpg1 cell;

B:Localization of ATF4 (red), CHOP (red), XBP1s (red), ATF6 (red) in CCPG1 overexpression granulosa cells

β-actin was used as a reference protein, ***$p < 0.001$, **$p < 0.01$, *$p < 0.05$.

Nuclei stained with DAPI (blue). Scale bar: 5 μm.

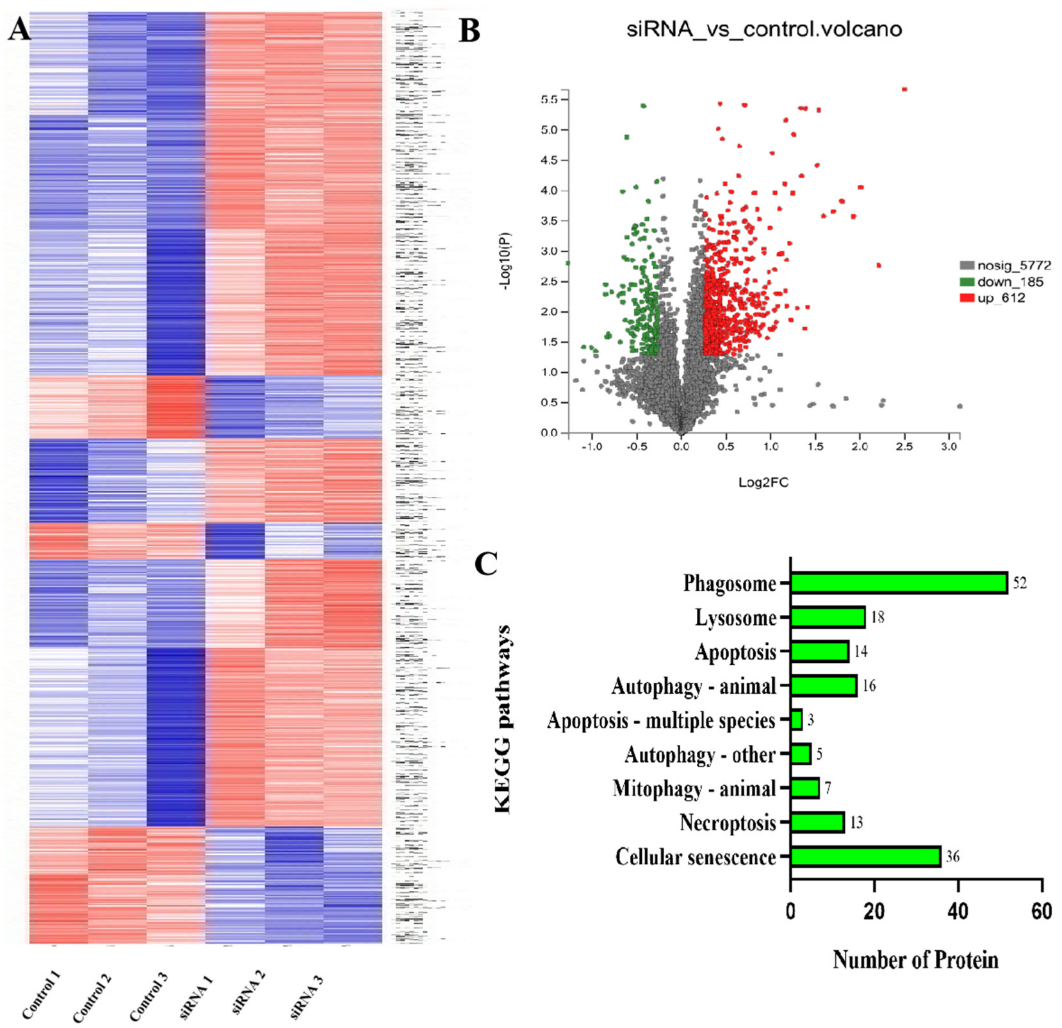

Figure S2. The results and analysis of proteome in ccpg1 knockdown and control cells

A: The heatmap of knockdown ccpg1; B: The volcano plots of down and up-regulated proteins; C: KEGG enrichment of cell death related pathway.

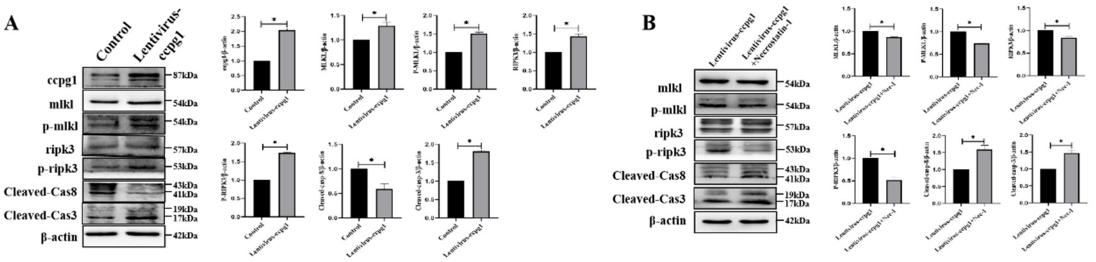

Figure S3. Activated necroptosis in up-regulated ccpg1 cells

A: Detection cell necroptosis by western blot and results in over-expression of ccpg1 cell;

B: Detection cell necroptosis by western blot and results in necroptosis inhibitor treated cells.

β-actin used as reference protein, ***$p < 0.001$, **$p < 0.01$, *$p < 0.05$

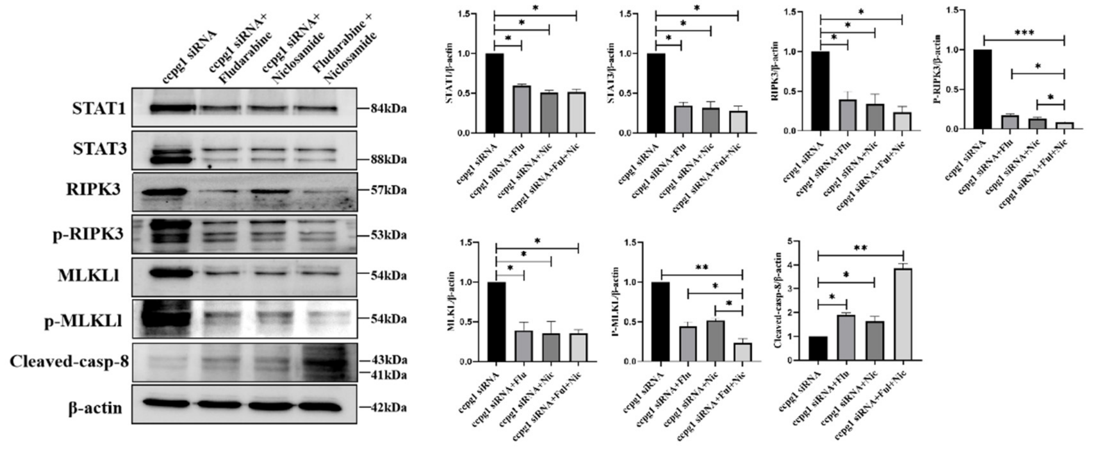

Figure S4. The necroptosis was coordinately regulated by STAT1 and STAT3

Detection cell necroptosis by western blot and results in siRNA ccpg1 treated cells, siRNA ccpg1+Fludrabine (STAT1 inhibitor) treated cells, siRNA ccpg1+Niclosamide (STAT3 inhibitor) treated cells, siRNA CCPG1+Fludrabine+Niclosamide treated cells

β-actin used as reference protein, ****p*< 0.001, ***p*< 0.01, **p*< 0.05

A

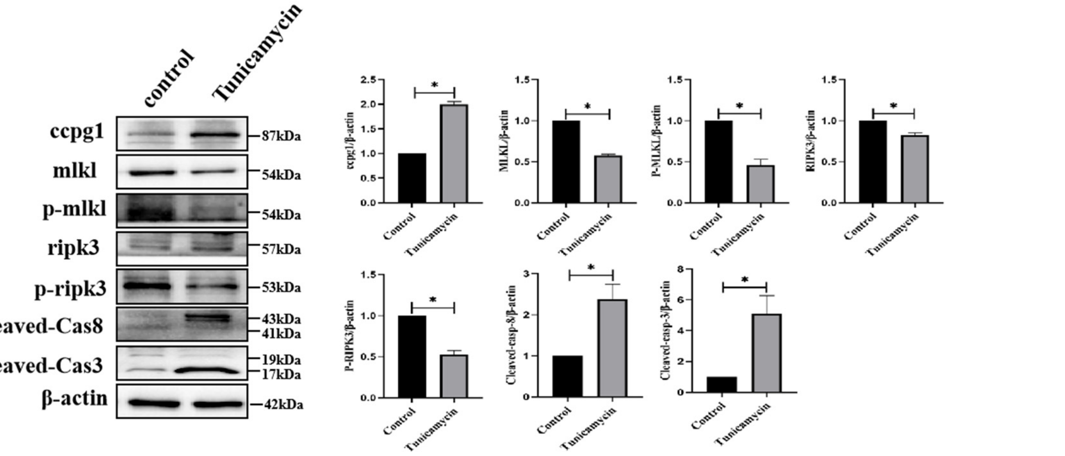

B

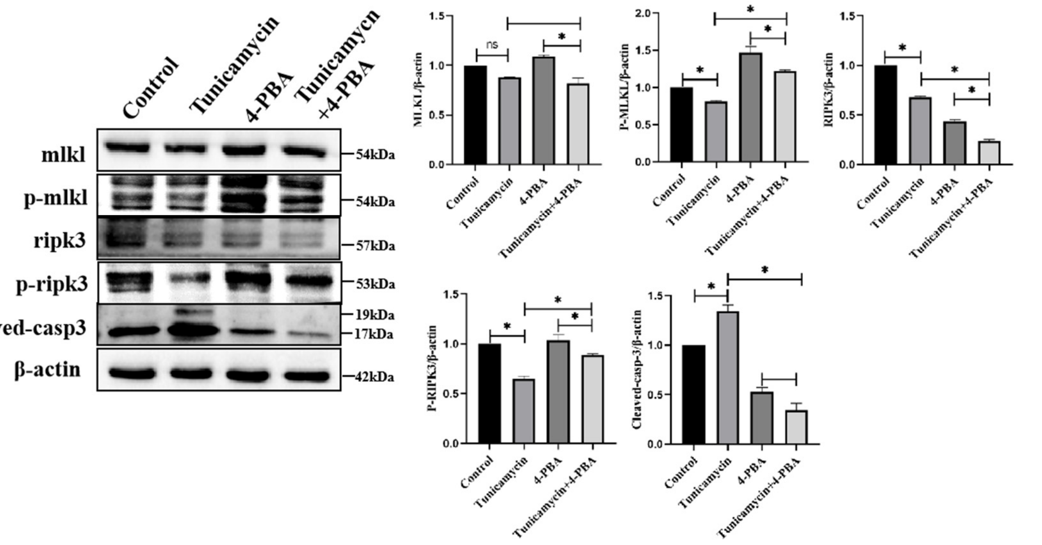

Figure S5. Activated necroptosis negatively related with ER stress mediated apoptosis

A: Detection cell necroptosis by western blot and results in tunicamycin treated and control cell;

B: Detection cell necroptosis by western blot and results in ER stress inhibitor 4-PBA, 4-PBA+tunicamycin, tunicamycin treated and control cells.

β-actin used as reference protein, ***$p < 0.001$, **$p < 0.01$, *$p < 0.05$
